# Clinical Pathology Reference Intervals for an In-Water Population of Juvenile Loggerhead Sea Turtles (*Caretta caretta*) in Core Sound, North Carolina, USA

**DOI:** 10.1371/journal.pone.0115739

**Published:** 2015-03-04

**Authors:** Terra R. Kelly, Joanne Braun McNeill, Larisa Avens, April Goodman Hall, Lisa R. Goshe, Aleta A. Hohn, Matthew H. Godfrey, A. Nicole Mihnovets, Wendy M. Cluse, Craig A. Harms

**Affiliations:** 1 Environmental Medicine Consortium, North Carolina State University, 1060 William Moore Drive, Raleigh, North Carolina 27607, United States of America; 2 Department of Clinical Sciences, North Carolina State University, 1060 William Moore Drive, Raleigh, North Carolina 27607, United States of America; 3 National Marine Fisheries Service, National Oceanic and Atmospheric Administration, Beaufort Laboratory, 101 Pivers Island Road, Beaufort, North Carolina 28516, United States of America; 4 North Carolina Wildlife Resources Commission, 1507 Ann St., Beaufort, North Carolina 28516, United States of America; 5 Department of Ecology, Evolution, and Environmental Biology, 1200 Amsterdam Avenue, Columbia University, New York, New York 10027, United States of America; 6 Center for Marine Sciences and Technology, 303 College Circle, Morehead City, North Carolina 27606, United States of America; USGS National Wildlife Health Center, UNITED STATES

## Abstract

The loggerhead sea turtle (*Caretta caretta*) is found throughout the waters of the Atlantic, Pacific, and Indian Oceans. It is a protected species throughout much of its range due to threats such as habitat loss, fisheries interactions, hatchling predation, and marine debris. Loggerheads that occur in the southeastern U.S. are listed as “threatened” on the U.S. Endangered Species List, and receive state and federal protection. As part of an on-going population assessment conducted by the National Marine Fisheries Service, samples were collected from juvenile loggerhead sea turtles in Core Sound, North Carolina, between 2004 and 2007 to gain insight on the baseline health of the threatened Northwest Atlantic Ocean population. The aims of the current study were to establish hematologic and biochemical reference intervals for this population, and to assess variation of the hematologic and plasma biochemical analytes by season, water temperature, and sex and size of the turtles. Reference intervals for the clinical pathology parameters were estimated following Clinical Laboratory Standards Institute guidelines. Season, water temperature, sex, and size of the turtles were found to be significant factors of variation for parameter values. Seasonal variation could be attributed to physiological effects of decreasing photoperiod, cooler water temperature, and migration during the fall months. Packed cell volume, total protein, and albumin increased with increasing size of the turtles. The size-related differences in analytes documented in the present study are consistent with other reports of variation in clinical pathology parameters by size and age in sea turtles. As a component of a health assessment of juvenile loggerhead sea turtles in North Carolina, this study will serve as a baseline aiding in evaluation of trends for this population and as a diagnostic tool for assessing the health and prognosis for loggerhead sea turtles undergoing rehabilitation.

## Introduction

The loggerhead sea turtle (*Caretta caretta*) is a marine turtle species that occurs throughout the temperate and tropical regions of the Atlantic, Pacific, and Indian Oceans [1]. In the United States and elsewhere, loggerheads face multiple and sometimes synergistic threats, including fisheries interactions, habitat loss, hatchling predation, marine pollution and debris, trauma, and disease [2]. Loggerhead sea turtles in the southeastern U.S. are listed as “threatened” on the U.S. Endangered Species List, and are protected by state and federal laws [3]. A recovery plan for loggerheads in this region was published in 2008, and included among the various actions are comprehensive population health and viability assessments [4]. Additional conservation initiatives involve improved medical management of stranded turtles undergoing temporary rehabilitation [4].

Assessment of hematological and biochemical parameters provides useful information for monitoring the health status of marine turtles [5,6]. For instance, Keller et al. (2004) documented correlations between organochlorine contaminant concentrations and various hematology and clinical blood chemistry values in loggerhead sea turtles in the waters of North Carolina, suggesting that organochlorines may negatively impact the health status of exposed sea turtles [7]. Furthermore, fibropapillomatosis, a neoplastic disease of marine turtles, has been found to alter hematological and biochemical values in affected individuals [5]. In addition to providing information that is valuable for monitoring population health, clinical health parameters serve as useful measures for evaluating the health status of stranded individuals, and can serve as indicators for monitoring progress in the rehabilitation process, including suitability for release back to the wild. Because hematologic and plasma biochemistry analytes vary significantly among loggerhead sea turtle populations in the wild, it is important to establish population-specific baseline information [8]. In order to monitor the status of in-water loggerhead sea turtle populations, as specified in the U.S. recovery plan [4], a cooperative research program between the National Marine Fisheries Service Beaufort Laboratory and commercial pound net fishers was initiated in Pamlico and Core Sounds, North Carolina, in 1995 [9]. This partnership has provided researchers with opportunities to sample large numbers of incidentally captured live loggerheads [9]. As part of this program, blood samples and other health-related data were collected from the turtles between 2004 and 2007 to improve understanding of population health and also establish baseline information for long-term monitoring. The present study fulfilled two objectives in the overall health assessment for the juvenile loggerhead sea turtle population in North Carolina: 1) establishing hematologic and plasma biochemical reference intervals, and 2) examining variation of the hematologic and biochemical parameters by season, water temperature, and sex and size of the loggerheads.

## Materials and Methods

### Ethics Statements

The Northwest Atlantic Ocean distinct population segment sampled for the current study is listed as threatened under the US Endangered Species Act [10]. Capture and sampling protocols were conducted under the authority of US Endangered Species Act Section 10(a)(1)(A) scientific research permits from the United States Fish and Wildlife Service (TE676379–5) and the National Marine Fisheries Service (1260), as well as North Carolina Wildlife Resources Commission permits (04ST63, 05ST63, 06ST63, 07ST63, 08ST63). All animal-related procedures were approved by the North Carolina State University Animal Care and Use Committee (04–092-O). Care was taken to minimize stress while handling the turtles. The capture technique utilized causes minimal disturbances in blood gases and acid-base balance [11].

### Reference Population and Study Site

**Fig 1:**
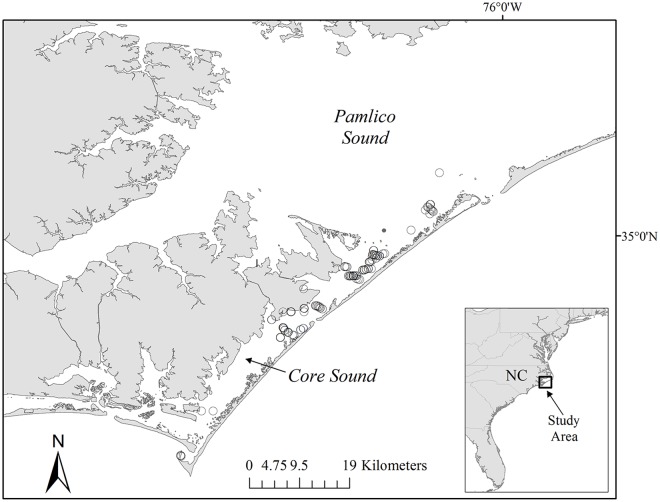
Capture sites within Core Sound, North Carolina where juvenile loggerhead sea turtles were sampled to estimate hematologic and biochemical reference intervals for this population.

The sounds of North Carolina serve as important foraging grounds for juvenile loggerhead sea turtles [12]. As water temperatures warm in the spring, loggerheads enter and disperse throughout the sounds [13]. While some turtles remain within these estuarine waters during warmer times of the year, others continue their migratory movement to more northerly foraging areas. As water temperatures start to cool in the fall, turtles emigrate from the sounds and overwinter in the warmer coastal waters off North Carolina or further south [12,14]. From 2004 to 2007, 205 juvenile loggerhead sea turtles were sampled from stationary pound nets utilized by commercial fishermen to capture flounder in Core Sound, North Carolina, U.S. (Fig. 1).

### Sample Collection and Laboratory Analysis

Samples for this study were collected between May and November when the pound net fishery is operational. For each sampling event, surface water temperature was recorded to the nearest 0.5°C using calibrated thermometers. Nets were fished and sea turtles retrieved every 3 to 4 days. The entrance/exit to the net remained open throughout deployment and the turtles could feed, swim, and surface to breathe while in the nets. Standard morphometric measurements were obtained from each turtle, including standard straightline carapace length to the nearest 0.1 cm [15]. Body weights could not be obtained in the small fishing boats. For each first capture, both rear flippers of each turtle were tagged with external Inconel flipper tags (National Band and Tag Company, Newport, KY) and a single passive internal transponder tag (Destron-Fearing Corp., South St. Paul, MN) was injected into the triceps muscle complex of the turtle’s left front flipper. A general physical examination was performed on each turtle and any individuals deemed to be unhealthy (e.g., thin, weak, lethargic, injured, heavy epibiota, external masses) were transported to a rehabilitation center for treatment and excluded from the analyses. With the exception of 2007, laparoscopy was performed on a subset of the turtles for identification of sex during two separate one-week periods in the summer and fall. This technique was used to validate serum testosterone concentrations as a comparatively non-invasive means of identifying sex in juvenile loggerhead sea turtles in different seasons for use in population monitoring and modeling [16]. The sex of the remaining individuals was identified by assessing serum testosterone levels when water temperature was ≤ 20°C.

Within 15 minutes of retrieval from the net, venous blood samples were collected from the dorsal cervical sinus into sodium heparin blood tubes (Monoject, Sherwood Medical, St. Louis, MO) using a 21 gauge Vacutainer (BD Vacutainer, BD, Franklin Lakes, NJ) needle with an associated plastic hub. The blood tubes were inverted gently several times to ensure proper mixing of blood and anti-coagulant and then were immediately placed on ice and processed within 5 to 6 hours, a time span associated with minimal clinical pathology alterations in refrigerated loggerhead sea turtle blood [17]. Whole blood and plasma were shipped the same day to Antech Diagnostic Laboratories (Antech Diagnostic Laboratories, Memphis, Tennessee, USA) for a complete blood count and biochemical profile (Antech’s Comprehensive Reptile Profile-AE160).

### Statistical Analysis

Reference intervals were estimated using data from 191 apparently healthy individual juvenile loggerhead sea turtles (103 females, 48 males, and 40 of unknown sex) collected over the four year period (S1 Table). Thirty-seven of these individuals were sampled more than once during the study. A random number generator was used to select entries to retain for individuals that had multiple measurements. In addition, for those individuals that underwent laparoscopy for identifying sex, and were later recaptured, subsequent clinical pathology measurements were excluded from the analysis to avoid including hematological or biochemical parameter values that may have been affected by the surgical procedure.

Histograms, boxplots, and normal probability plots were used to assess distributions of each variable. Normality was tested for using the D’Agostino-Pearson test, and natural logarithmic and Box Cox transformations were performed on variables that were not normally distributed. Transformed data for a number of analytes did not conform to a Gaussian distribution; therefore the non-parametric method was used for reference interval estimation. In addition to visual examination of boxplots and histograms, outliers were identified using the Dixon-Reed outlier test [18] and were manually removed from reference interval estimation. The 95% reference intervals and associated 90% confidence intervals (CIs) for the limits of each interval were estimated using the non-parametric method following Clinical Laboratory Standards Institute (CLSI) C28-A3 guidelines (2.5–97.5 percentiles) [19].

Because seasonal variation in clinical pathology parameters has been previously identified in inshore juvenile loggerhead sea turtles in North Carolina [20], the hematological and biochemical data were assessed for utility of partitioning the reference intervals into subclasses based on season [19]. In addition, some analytes, such as uric acid and cholesterol, vary by sex in marine turtles [21], so analyte data were also assessed for partitioning into subclasses based on sex. Data were grouped for comparison by season according to sampling periods. The summer sampling period extended from the beginning of May through the end of September when the loggerhead sea turtle population in Core Sound is thought to consist primarily of resident turtles (n = 113). The fall sampling period occurred during October and November (n = 78) when individuals are migrating through the estuary and the population in Core Sound consists of both resident and migrant loggerhead sea turtles [12,22,23].

While there is no consensus on the criteria to be used for determining whether partitioning is pertinent [19,24,25], the CLSI C28-A3 guidelines recommend consideration of physiological differences that are expected to result in important clinical differences in the reference intervals in addition to analytical methods [19]. Statistical tests are often utilized to compare analyte data between subclasses and justify partitioning of the reference intervals, however the CLSI guidelines discuss the limitation that small differences between subclasses may be statistically significant, but lack clinical importance. Therefore, the guidelines recommend the use of other methods such as proportion criteria (i.e., criteria that relate to proportions of the subgroups outside each of the reference limits of the combined distribution) [24] as an analytical tool to aid in assessment of the need for partitioning [19]. Clinical pathology analytes presumed to have important physiological differences by season and sex in this study were assessed for partitioning into subclasses using the proportion criteria as described previously [24]. Because a minimum of 120 samples in each subclass is necessary to achieve a 90% confidence interval using nonparametric methods, the robust method following CLSI C28-A3 guidelines [19] was used to estimate the reference interval values for partitioned analytes.

The hematology and biochemistry data were assessed for significant differences by sex and season using the Wilcoxon rank sum test and by size (i.e., straightline carapace length) and water temperature using the Spearman rank correlation coefficient. In addition, seasonal variation in the sex and size of turtles was evaluated using the Fisher’s exact test. Reference intervals were estimated using MedCalc Software bvba version 13.2 (Mariakerke, Belgium). All other analyses were performed in R version 2.15.3 (Vienna, Austria) [26].

## Results

No external masses consistent with fibropapillomatosis were observed on any of the loggerhead turtles captured for this study. The straight carapace length of the sampled juvenile loggerhead sea turtles ranged from 50.4 to 80.6 cm, with a median of 63.5 cm. The water temperature during the sampling events was 14.5–31°C (median = 26°C) during the summer sampling period and from 9–26°C (median = 22°C) during the fall sampling period.

Hematologic and serum biochemistry reference intervals for this population of juvenile loggerheads are presented in Table 1. A total of 8 unhealthy turtles were excluded from the analyses. In addition, blood measurements from 6 turtles were omitted from the analyses because of significant outliers identified using the Dixon-Reed outlier test in 3 turtles and missing data for more than one analyte in another 3 individuals. Blood urea nitrogen was the only analyte that met the proportion criteria for partitioning into reference interval subclasses based on seasonal sampling period, and none of the analytes met the criteria for partitioning by sex. However, a number of the hematologic and biochemical parameters did exhibit lesser but statistically significant variation based on season (Table 2). The total estimated white blood cell, heterophil, and monocyte counts, total protein, globulin, and blood urea nitrogen were significantly higher in turtles sampled during the fall compared to the summer sampling period. Packed cell volume and chloride were significantly lower in turtles sampled during the fall period relative to turtles sampled during the summer period. A number of clinical pathology parameters were correlated with water temperature (Table 3). Total estimated white blood cell count, heterophil count, phosphorous, and CPK were negatively correlated with water temperature. Clinical pathology parameters that were positively correlated with water temperature include packed cell volume, glucose, AST, calcium, potassium, and uric acid.

**Table 1:** Hematology and plasma biochemistry reference intervals and 90% confidence intervals for a population of 191 apparently healthy juvenile loggerhead sea turtles (*Caretta caretta*) sampled in Core Sound, North Carolina, USA from 2004–2007.

Parameter	Unit	N^a^	Median (Range)	Lower limit (90% CI)	Upper limit (90% CI)
Packed cell volume	%	190	31.0 (9.0–40.0)	17.0 (10.0–20.0)	39.0 (38.0–40.0)
Estimated white blood cell count	X 10^9^/L	190	9.0 (2.0–27.0)	5.0 (2.0–5.0)	19.0 (16.0–27.0)
Heterophils	X 10^9^/L	190	4.7 (0.0–21.6)	0.0 (0.0–0.8)	13.6 (12.2–16.1)
Lymphocytes	X 10^9^/L	190	3.4 (0.6–9.2)	1.0 (0.7–1.1)	7.9 (7.2–9.2)
Monocytes	X 10^9^/L	190	0.14 (0.0–1.6)	0.0 (0.0–0.0)	1.0 (0.8–1.6)
Eosinophils	X 10^9^/L	190	0.3 (0.0–4.8)	0.0 (0.0–0.0)	2.9 (1.6–4.0)
Basophils	X 10^9^/L	190	0.0 (0.0–0.5)	0.0 (0.0–0.0)	0.3 (0.2–0.4)
Azurophils	X 10^9^/L	190	0.0 (0.0–1.2)	0.00 (0.0–0.0)	0.63 (0.5–1.2)
Total protein	g/L	191	35.0 (21.0–60.0)	22.8 (22.0–24.0)	52.2 (47.0–53.0)
Albumin	g/L	191	11.0 (4.0–17.0)	5.0 (5.0–6.0)	16.0 (16.0–17.0)
Glucose	mmol/L	191	5.8 (2.5–12.9)	3.7 (3.1–4.1)	9.1 (8.3–10.2)
Aspartate aminotransferase	U/L	191	161.0 (50.0–390.0)	84.4 (76.0–93.0)	304.8 (269.0–362.0)
Calcium	mmol/L	191	1.9 (1.3–2.9)	1.4 (1.3–1.5)	2.5 (2.4–2.6)
Phosphorus	mmol/L	191	2.2 (1.2–3.6)	1.4 (1.3–1.6)	3.3 (3.1–3.6)
Calcium/phosphorus ratio	mmol/L/mmol/L	191	0.8 (0.4–1.8)	0.5 (0.4–0.6)	1.4 (1.3–1.6)
Sodium	mmol/L	191	156.0 (145.0–168.0)	150.0 (145.0–150.0)	163.0 (162.0–165.0)
Potassium	mmol/L	191	4.2 (2.5–6.1)	2.9 (2.5–3.1)	5.2 (5.1–5.7)
Chloride	mmol/L	191	115.0 (101.0–129.0)	103.0 (102.0–107.0)	125.0 (123.0–125.0)
Globulin	g/L	191	24.0 (13.0–46.0)	14.0 (14.0–16.0)	37.0 (33.0–38.0)
Creatine phosphokinase	U/L	191	1,034.0 (153.0–13,310.0)	249.4 (165.0–297.0)	4409.0 (3782.0–5123.0)
Uric acid	μmol/L	191	47.6 (5.9–166.5)	6.0 (6.0–11.9)	142.8 (113.0–142.8)
**Reference interval partitioned based on season**					
Blood urea nitrogen	mmol/L				
Fall sampling period		78	33.7 (11.8–62.8)	13.1 (11.0–15.7)	61.3 (56.5–65.9)
Summer sampling period		113	23.6 (6.1–67.8)	7.2 (6.1–8.6)	63.9 (56.8–71.0)

^a^Sample sizes varied among analytes for reference interval estimation due to missing data for a few hematological analytes.

**Table 2:** Hematology and plasma biochemical parameters that exhibited significant variation between the fall (October and November) and summer (May through September) sampling periods in juvenile loggerhead sea turtles (*Caretta caretta*) sampled in Core Sound, North Carolina, USA.

Parameter	Fall Median	Summer Median	P-value
Estimated white blood cell count (X 10^9^/L)	10	9	< 0.0001
Heterophils (X 10^9^/L)	6.4	4.1	< 0.0001
Monocytes (X 10^9^/L)	2.8	1.0	0.0001
Total protein (g/L)	37	33	0.0020
Globulin (g/L)	26	22	0.0040
Blood urea nitrogen (mmol/L)	34	24	0.0001
Packed cell volume (%)	29	31	0.0003
Chloride (mmol/L)	114	116	0.0300

Only blood urea nitrogen differences met criteria for partitioning by season for reference intervals.

**Table 3:** Hematology and plasma biochemical parameters that were significantly correlated with water temperature in juvenile loggerhead sea turtles (*Caretta caretta*) sampled in Core Sound, North Carolina, USA.

Parameter	Correlation with Water Temperature	Spearman’s ρ	P-value
Estimated white blood cell count	−	-0.18	0.010
Heterophils	−	-0.32	< 0.001
Phosphorous	−	-0.20	0.005
Creatine phosphokinase	−	-0.28	< 0.001
Packed cell volume	+	0.40	<0.001
Glucose	+	0.22	0.002
Aspartate aminotransferase	+	0.44	<0.001
Calcium	+	0.24	0.001
Potassium	+	0.49	<0.001
Uric Acid	+	0.17	0.020

Assessment for differences in clinical pathology parameters by sex revealed that glucose was higher in males than females (P = 0.03). A number of parameters were positively correlated with straight carapace length, including packed cell volume (Spearman’s ρ = 0.30; P = 0.004), total protein (Spearman’s ρ = 0.25; P = 0.02), albumin (Spearman’s ρ = 0.29; P = 0.006), and phosphorus (Spearman’s ρ = 0.40; P = 0.0001). There were no significant differences in the size and sex distribution of turtles sampled for this study by season.

## Discussion

### Reference intervals

Establishing reference intervals to be used for health assessment of individuals or populations requires a clinically normal sample that is most representative of the target population with regard to health, age and sex distribution, geography, and biology [27]. For this reason, there have been a number of surveys reporting reference values and intervals for different species of sea turtle populations around the world. In comparison to a number of hematologic and biochemistry reference intervals published for free-ranging marine turtle populations, the intervals for this loggerhead sea turtle population were estimated from a relatively large sample of turtles. In fact, the sample size of 191 individuals exceeded the CLSI guidelines of 120 individuals to estimate reference values using nonparametric methods from non-Gaussian distributed data and to estimate partitioned reference values using robust methods from transformed data (i.e., blood urea nitrogen). Only one other study reporting reference intervals for hematology and plasma biochemistry analytes in free-ranging turtles (i.e., green turtles (*Chelonia mydas*) in Australia) has met this sample size criterion for nonparametric estimation [28]. Unlike for domestic animals and humans, estimating reference intervals for free-ranging wildlife is challenging due to small numbers of individuals, limitations in accessing individuals for sampling, and constrained resources. Because of these challenges, long-term studies are needed to ensure opportunities to establish valuable baseline health information with large enough sample sizes, including clinical pathology reference intervals, which can be used for assessing temporal patterns and in detecting changes in health status among sea turtle populations.

Comparison of clinical pathology reference intervals established for this study with values and intervals reported for other free-ranging loggerhead sea turtle populations was limited by different methodology used by investigators to generate the intervals. Despite these differences, hematologic reference intervals estimated for loggerheads in this study, with few exceptions, are in accordance with those documented for other free-ranging loggerhead sea turtle populations [8,20,29]. The lower limit for packed cell volume in the reference interval estimated for loggerheads in this study is lower compared to the limits reported previously for apparently healthy juvenile loggerheads sampled in Core and Pamlico Sounds, North Carolina (lower limit for comparison = 10th percentile) [20] and along the coast of Georgia (lower limit for comparison = mean—2 standard deviations) [29]. However, the lower limit for packed cell volume is similar to that reported for free-ranging clinically healthy large immature and mature loggerhead sea turtles sampled off the coast of Australia [8]. In addition, loggerheads sampled for this study have wider reference intervals for estimated white blood cell, heterophil, and lymphocyte counts relative to the loggerheads sampled in the Georgia coastal waters [29].

Similarly, there are few differences between plasma biochemistry reference intervals estimated for loggerheads in this study and those reported in the aforementioned studies [8,20,29]. The lower limits of the reference intervals for total protein and albumin estimated for this sample of loggerhead turtles are lower than those previously reported in North Carolina and Georgia [20,29]. However, the reference intervals generated for total protein and albumin for the loggerheads sampled in Australia are similar to those reported here [8]. Globulin and blood urea nitrogen concentrations were more variable among loggerheads sampled for this study, as evidenced by wider reference intervals relative to concentrations reported for the previous studies conducted in North Carolina and Georgia. In addition, in comparison to reference values reported in prior years in North Carolina, the reference values for uric acid are lower than the previously reported values, and the upper limit for sodium is higher than the previously reported 90th percentile for that analyte [20]. The reference intervals for glucose, potassium, and chloride are similar among studies.

Differences between the current results for packed cell volume, total protein, albumin, globulin, BUN, uric acid and sodium, and those from a previous study of the same population [20] may be an artifact of smaller sample sizes and different seasonal balance in that study (n = 42 in November of 1997 and n = 15 in August of 2000). Alternatively, the differences could reflect real differences in population health suggestive of subtle decline in general health or nutritional status and homeostasis in clinical pathology analytes in the intervening 4 to 10 year period. These two studies mark the beginning of a data series for monitoring long term trends of health indicators in relation to population size, forage quality, offshore and near shore energy development, and climate conditions.

Sampling wild populations to establish hematology and plasma biochemistry reference intervals [8,21,28,29] provides benchmarks for evaluating health status of individual animals under treatment in rehabilitation or aquarium care. Typically no interpretive guidance is provided beyond aiming between upper and lower limits as established, in concert with an overall assessment of body condition, behavior and swimming ability. However, some of the resulting reference limits (e.g., packed cell volume of 13.9% and albumin of 6 g/L [8], packed cell volume of 17% and albumin of 5 g/L in the present study) fail to meet standards for convalescent sea turtles for some rehabilitation facilities [30,31], or for healthy captive-reared [32] or wild [33] loggerhead turtles of comparable size. Possibilities for these discrepancies include excessively stringent pre-release standards in some rehabilitation facilities, or an imperfect visual assessment in the field of unhealthy turtles as healthy. Without discounting either possibility, a reasonable approach to assessing release status for a rehabilitated sea turtle might be to aim closer to the median than the tail values of the wild population. This approach may help ensure adequate health reserves to cope with readjustment to wild conditions. Conversely, hematology and plasma biochemistry values exacerbated by captive conditions, such as markedly inverted calcium/phosphorus ratios due to unavailability of natural diets [30] may be criteria for accelerating release. The calcium/phosphorus ratio is commonly evaluated in reptile medicine to assess mineral balance with respect to nutritional and renal status, with a value greater than 1:1 (on a mg/dL conventional unit basis, equivalent to 0.774:1 in standard international (SI) units) considered optimal [34]. At least slightly inverted calcium/phosphorus ratios were present in nearly 50% of the current study population, however, indicating this criterion is not strictly applicable in loggerhead turtles. Degree of calcium/phosphorus ratio inversion becomes concerning as it approaches the lower limit (0.51 SI unit, 0.66 conventional unit).

### Seasonal and water temperature related variation in analytes

With the exception of blood urea nitrogen, the clinical pathology parameters did not meet the criteria for partitioning by season for the purpose of calculating separate reference intervals. However, several analytes did exhibit statistically significant differences by season. Juvenile loggerheads sampled during the fall for this study had higher estimated white blood cell and heterophil counts relative to turtles sampled during the summer. In addition, there was a negative correlation between water temperature and the estimated white blood cell and heterophil counts. Increased numbers of granulocytes can indicate a stress leukogram in reptiles [35,36], and could reflect a stress response by the loggerheads to decreasing photoperiod and cooler temperatures in the fall.

As water temperatures cool during the fall months, there is an influx of loggerhead sea turtles from northern areas into NC waters, as they migrate southward along the coast to warmer areas [12,14]. Throughout migration, sea turtles are exposed to many physiologic stressors including changes in temperature, salinity, and food availability [20]. There was not a mechanism to discern between resident and migrant loggerhead turtles in this study; therefore, variation in clinical pathology parameters by migratory status could not be assessed directly. The higher estimated white blood cell and heterophil counts in turtles sampled during the fall months may be partly explained by migrants within the sample of loggerheads in the fall. A previous survey of juvenile loggerhead sea turtles in Core and Pamlico Sounds in North Carolina reported a relatively higher percentage of heterophils in migratory turtles compared to residents [20]. The migrants also had lower lymphocyte and eosinophil counts providing further evidence of a stress leukogram in this group of turtles [20]. There was no seasonal variation in lymphocyte or eosinophil counts in the current study.

Granulocyte counts also increase with inflammatory processes, including infectious disease [35,36]. Although the loggerheads were apparently healthy, it is possible that turtles sampled during the fall months had a higher incidence of underlying inflammatory disease relative to turtles sampled during the summer. This is also supported by the higher monocytes and globulin values [35,36] exhibited in turtles sampled during the fall compared to the summer.

In addition, loggerheads sampled in October and November had lower packed cell volume values, which was consistent with patterns documented for the juveniles previously sampled in the sounds of North Carolina [20]. The significantly lower packed cell volume in turtles sampled during the fall period compared to those sampled during the summer, and the positive correlation between packed cell volume and water temperature could be explained by seasonal variation in food consumption and assimilation. Sea turtles decrease their food intake and have less efficient digestion during cooler water temperatures and decreasing light [37]. Reptiles also have a slower rate of erythropoiesis during colder weather potentially contributing to the lower packed cell volume in turtles sampled during the fall [38].

Blood urea nitrogen concentrations were higher in loggerheads sampled during the fall relative to the summer. Although the reason for the seasonal variation is unknown, elevations in blood urea nitrogen could be due to dehydration or muscle catabolism [35] associated with migration following a summer season of foraging and growth. The negative correlation between water temperature and creatine phosphokinase concentrations in turtles sampled for the current study lends further support to physiological effects of muscle catabolism in turtles sampled during the fall. Furthermore, chloride values were lower in turtles sampled during the fall compared to the summer. Because the main source of chloride for sea turtles is through their diet and seawater consumed during foraging [20], variation in chloride concentrations could be the result of seasonal differences in food consumption and assimilation. In addition, there were positive correlations between water temperature and glucose, uric acid, calcium, and potassium concentrations. These clinical pathology analytes could similarly be influenced by variations in foraging and saltwater consumption that can occur with differences in temperature [35]. These results are consistent with Stamper et al., who documented lower glucose, sodium, chloride, and potassium concentrations in migrant loggerheads sampled in the fall in the sounds of North Carolina suggesting less food intake relative to resident turtles [20]. Because the size and sex of turtles sampled for this study were not variable by season, variation in the clinical pathology parameters was not likely confounded by differences in the size or age, and sex of the turtles.

### Sex-related variation in analytes

With the exception of higher glucose values in males, clinical pathology analytes of loggerheads sampled for this study did not differ significantly by sex. The lack of variation was likely the result of sexual immaturity of loggerheads sampled for this study, and therefore relative similarities in biology and physiology between males and females. Differences in clinical pathology by sex have been documented for some analytes (e.g., uric acid and cholesterol) in immature sea turtles [21], but most variation has been reported for parameters in adult sea turtles (e.g., albumin, calcium, cholesterol, triglycerides, phosphorus, and glucose) and has been suggested to arise from vitellogenesis in the females and differences in foraging during the breeding cycle [39–41].

### Size-related variation in analytes

Size and age related variations in clinical pathology analytes have been documented in a number of studies assessing hematologic and biochemical parameters in captive and wild sea turtles [32,40,42–45]. The significant positive correlation between size and packed cell volume in these loggerheads is consistent with previous reports of increasing packed cell volume with age and size in both captive and wild sea turtles [32,42–46]. In addition, significant increases in total protein and albumin with increasing size and age have also been observed in a number of studies investigating clinical pathology in sea turtles [21,32,40,42,44,47]. The reason for increasing phosphorus concentrations with increasing size of the turtles is unknown, but could be related to shifts in diet as the turtles grow larger.

## Conclusion

The use of clinical pathology to evaluate sea turtle health has important applications for rehabilitation of ill and injured sea turtles as well as for assessment of the health status of free-ranging sea turtle populations. Reference intervals can provide important information for evaluating the health of a particular population of turtles, serve as baseline information for monitoring the impacts of various disturbances on the population, guiding management of clinical cases in the rehabilitation setting, and establishing criteria for evaluating the prognosis for release of turtles from rehabilitation centers. Clinical pathology parameters can be variable based on a variety of factors, including age, size, sex, diet, season, and concurrent disease [5,20,21,33,44,48], therefore reference intervals specific to a particular population and partitioned into subgroups based on clinically relevant differences are important for the use of hematology and biochemistry as a diagnostic and prognostic tool for assessing individual and population health. Because loggerhead sea turtles are threatened, population health assessments are valuable for conservation and management. Our study has established an important frame of reference for conservation efforts dedicated to the recovery of the distinct population segment of loggerhead sea turtles inhabiting Core Sound, North Carolina.

## Supporting Information

S1 Table Demographic and clinical pathology data for juvenile loggerhead sea turtles sampled for this study. (XLS)
